# Aortic Unfolding Determined Using Non-Contrast Cardiac Computed Tomography: Correlations with Age and Coronary Artery Calcium Score

**DOI:** 10.1371/journal.pone.0095887

**Published:** 2014-04-22

**Authors:** Ji Won Lee, Jin Hur, Young Jin Kim, Hye-Jeong Lee, Ji Eun Nam, Hee-Yeong Kim, Yoo Jin Hong, Seok Min Ko, Tae Hoon Kim, Byoung Wook Choi

**Affiliations:** 1 Department of Radiology, Pusan National University Hospital, Pusan National University School of Medicine and Medical Research Institute, Busan, Republic of Korea; 2 Department of Radiology, Department of Cardiovascular Radiology, Research Institute of Radiological Science, Yonsei University College of Medicine, Seoul, Republic of Korea; 3 Department of Radiology, Kangwon National University Hospital, Chuncheon-Si, Gangwon-do, Korea; University of Groningen, Netherlands

## Abstract

**Objective:**

Aortic unfolding occurs with aging and reflects proximal aortic dilation, aortic arch widening, and decreased curvature. This study 1) evaluated the relationship between aortic unfolding measured using non-contrast cardiac-gated computed tomography (CT) and age, 2) assessed factors influencing aortic unfolding, and 3) determined the association of this measurement with coronary artery calcium (CAC) score.

**Methods:**

We reviewed the charts of 219 subjects (142 men, 77 women; mean age 54.2±9.3 years) who underwent coronary artery calcium scanning during routine health screening from December 2010 to May 2011. Multivariate regression analysis according to cardiovascular risk factors was performed. We also analyzed the relationship between aortic unfolding measurements and CAC score using stepwise multiple linear regression.

**Results:**

Mean aortic unfolding was 103.7±13.9 mm (men, 106.5±13.5 mm; women, 98.4±12.9 mm). Age, body surface area, and hypertension were exclusively associated with aortic unfolding. The association between aortic unfolding and CAC score was significant after adjustment for age and gender (β = 1.89, *p* = 0.017) and for Framingham risk score (β = 2.83, *p*<0.001).

**Conclusions:**

Aortic unfolding defined by measuring aortic width was a reproducible and practical method with non-contrast cardiac CT and associated with age, body surface area, and hypertension. CAC score, a well-established surrogate marker of cardiovascular disease, is positively associated with aortic unfolding. Further study to evaluate aortic unfolding as a potential predictor of cardiovascular risk is warranted.

## Introduction

Arterial changes with aging include lumen enlargement, wall thickening, and a reduction in the elasticity of large arteries, a process known as arteriosclerosis [1]. Although the principal change in arterial aging is medial degeneration, structural and functional changes also occur. The structural changes include aortic lumen enlargement and wall thickening, and these can be measured using ultrasound, invasive angiography, computed tomography (CT), or magnetic resonance imaging (MRI) [2]. Functional changes such as arterial stiffness reflecting a reduction in elasticity can be measured using several non-invasive methods, including pulse wave velocity (PWV) and the augmentation index (AI) [3], [4]. PWV is a powerful independent predictor of mortality in subjects with hypertension, end-stage renal failure, or diabetes, and was associated with higher cardiovascular mortality, coronary heart disease (CHD) events, and stroke in a community-dwelling sample of older people [5]–[7]. The carotid AI is an independent predictor of all-cause and cardiovascular mortality in patients with end-stage renal failure and an independent risk marker for premature coronary artery disease (CAD) [8]. However, the influence of blood pressure (BP) and heart rate are well-known limitations of PWV and AI determinations [9]–[11].

Aortic unfolding is a term used to describe the radiological abnormality on chest radiographs seen as widening of the mediastinum. This change occurs with aging and generally reflects proximal aortic dilation, aortic arch widening, and decreased curvature. Unfolding is often associated with aortic calcification which implies aortic degeneration and hypertension [2], [12]. Changes in the aortic dimensions are associated with the aortic PWV and AI between the aorta and brachial artery [13], [14]. Age-related changes in aortic arch geometry are related to functional aortic alterations, such as decreased aortic distensibility, augmented aortic arch PWV, and increased LV mass, in individuals without overt cardiovascular disease [15]. In the present study, we defined aortic unfolding as the width of the aorta which was measured on non-contrast electrocardiography (ECG)-synchronized cardiac CT, as a potential risk factor reflecting arterial aging. Measurements based on aortic width take advantage of a scale larger than those of lumen enlargement, wall thickening, or aortic distensibility, and are not influenced by temporary changes in BP or heart rate.

We evaluated aortic unfolding as a potential index of arterial aging by examining its correlation with age. In addition, to characterize aortic unfolding as a potential marker of individual cardiovascular risk, we assessed the factors influencing aortic unfolding based on correlations with risk factors for accelerated vascular aging and CAD. Finally, we analyzed the relationship between aortic unfolding measurements and coronary artery calcium (CAC) as a surrogate marker of coronary atherosclerosis burden and cardiovascular risk.

## Methods

### Study Participants

Our institutional review board (Yonsei University Health System) approved this study and waived informed consent for this retrospective review. We enrolled 229 consecutive asymptomatic, self-referred patients who underwent CAC scanning for screening purposes between December 2010 and May 2011 at our institution. Thoracic aortic aneurysm, severe pulmonary disease causing atelectasis or fibrosis around aorta, or kyphosis, which could influence aortic dimension, were not found on the plain chest radiographs in the subjects. We retrospectively reviewed the medical records of all subjects to collect clinical information, including demographic data and risk factors for coronary artery disease. Four subjects were excluded because of incomplete clinical information and six subjects were excluded due to known clinical CAD. Therefore, 219 subjects were analyzed in this study.

### CT Imaging Protocol

The subjects underwent CAC scanning with a 64-detector CT scanner (LightSpeed VCT; GE Healthcare, Waukesha, WI). A non-enhanced prospective ECG-gated axial scan was performed at 70% of the R-R interval with the following parameters: rotation time, 350 ms; section collimation, 0.625 mm×64; section width, 2.5 mm; tube voltage, 120 kV; and effective tube current–time product, 200 mAs. The scanning direction was craniocaudal, extending from the inferior border of the aortic arch to the bottom of the heart.

Using semi-automated software (AW Volume Share 4; GE Healthcare, Milwaukee, WI), coronary artery calcium was identified as a high-attenuation area, with attenuation exceeding the threshold of 130 HU, in the coronary artery. CAC scores were calculated according to the Agatston scoring method [16].

### Measuring Aortic Unfolding using Non-contrast Cardiac CT

**Figure 1:**
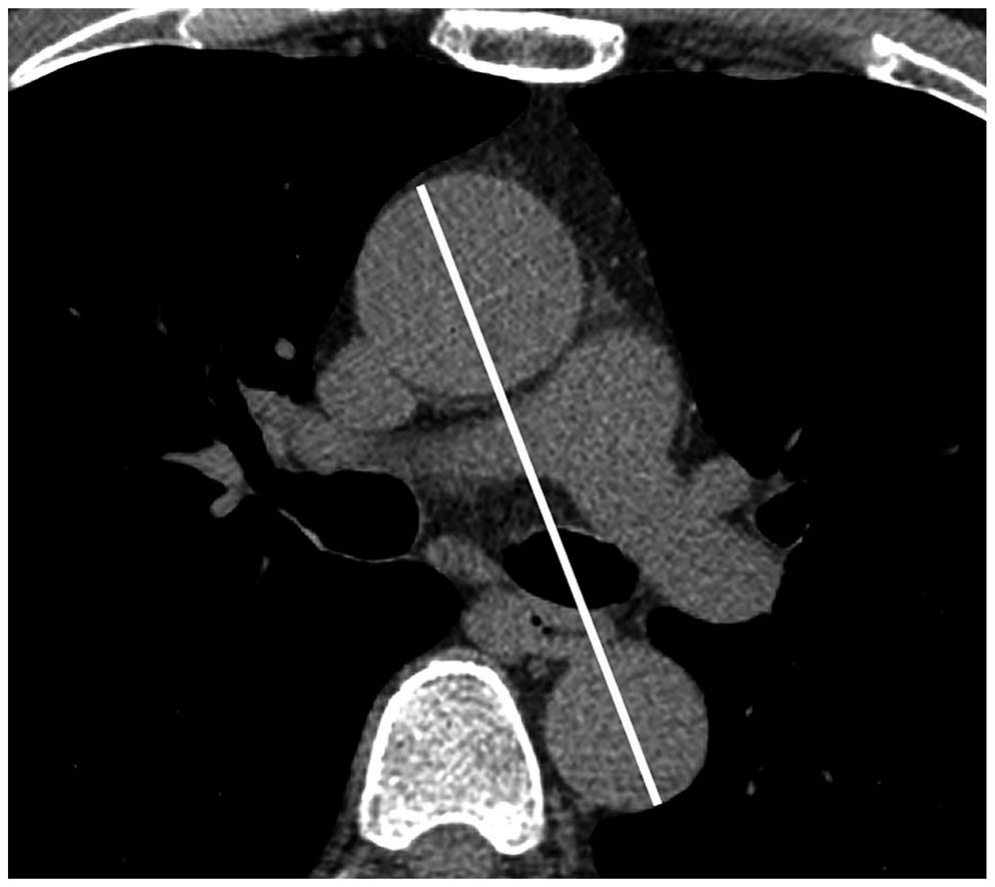
Measuring aortic unfolding. Aortic unfolding (white line) was defined as the longest distance between the ascending and descending aorta on a transaxial slice at the level of the pulmonary artery bifurcation on a selected coronary artery CT image.

Aortic unfolding was defined as the longest distance between the ascending and descending aortas, including the aortic lumen and aortic wall, on a selected CT slice at the level of the pulmonary artery bifurcation (Fig. 1). Two radiologists who were blind to the clinical information measured the aortic unfolding. To assess the intra-observer variability, each reader made two measurements in random order at an interval of at least 4 weeks. The mean value of the two measurements by the two radiologists was used for the analyses. Aortic unfolding index was defined as aortic unfolding divided by body surface area (BSA).

### Data Collection

Information on the presence of categorical cardiac risk factors was collected for each subject from a chart review [12], [17]–[19]. Body mass index (BMI) and BSA were calculated using the Mosteller method [20]. A family history of CAD was defined as myocardial infarction (MI), coronary revascularization, or sudden cardiac death occurring in a first-degree relative (men, age <55 years; women, age <65 years). The CT scan and ECG were performed within 7 days. Left ventricular hypertrophy (LVH) was defined as a Sokolow-Lyon index on the ECG: sum of SV_1_ plus RV_5_ or RV_6_ ≥35 mm [21]. Hypertension was defined as systolic BP ≥140 mm Hg, diastolic BP ≥90 mm Hg, or the use of antihypertensive medication. Subjects were classified as having diabetes if they had an established diagnosis of diabetes mellitus made by a physician, were being treated with insulin or oral hypoglycemic agents, or had measured fasting glucose ≥126 mg/dl. Dyslipidemia was defined as a total/high-density (HDL) cholesterol ratio >5 or the use of a hypocholesterolemic drug (statins or fibrates). Blood samples for lipid, fasting glucose, and plasma creatinine were obtained on the day of the CT examination. Positive smoking status was defined as current smoking or a history of smoking. Major adverse cardiac events information was obtained from patient telephone interviews and hospital records between January and April 2013.

### Framingham Risk Estimates

Of the 219 subjects, 212 subjects ranged in age from 30 to 74 years, corresponding to the age range of the Framingham cohorts. The 10-year risk of cardiovascular disease (CVD) in this group were calculated using the equations derived from the Framingham Heart Study and Framingham Offspring Study [22].

### Statistical Analyses

All statistical analyses were performed using SAS ver. 9.2 (SAS Institute, Cary, NC) and MedCalc ver 12.3.0.0 (MedCalc Software, Mariakerke, Belgium). For graphing, we used MedCalc ver 12.3.0.0 (MedCalc Software, Mariakerke, Belgium) and Excel 2007. The clinical characteristics of the study sample are reported as the mean±SD or the number and percentage. Continuous variables were compared using the *t-*test for two groups. Categorical variables were compared using Pearson's chi-squared statistic. The Jonckheere–Terpstra test was used to assess the trends across age groups. Univariate and multivariate regression analyses were performed according to sex, age, calcium score, BMI, BSA, family history of CAD, hypertension, diabetes mellitus, LVH on ECG, plasma creatinine, dyslipidemia, and smoking status. In the group of 212 patients corresponding to the Framingham cohort age range, Pearson's correlation coefficient was used to analyze the association between 10-year CVD risk and aortic unfolding. Stepwise multiple linear regression analysis was performed to assess the relationship between aortic unfolding and CAC score. Intra- and inter-observer reproducibility was calculated using the intra-class correlation coefficient (ICC), where ICC <0.4 represented poor reliability; ICC between 0.4 and 0.75, fair-to-good reliability; and ICC >0.75, excellent reliability. A value of *p*<0.05 was considered to indicate significance.

## Results

Table 1 shows the clinical characteristics of the subjects. Their ages ranged from 33 to 82 years, with a mean of 54.2±9 years [53.2±8.7 in men (n = 142) and 56.1±10.2 in women (n = 77)]. Men differed significantly from women for all parameters except family history of CHD, prevalence of hypertension, and dyslipidemia. In 219 subjects, the mean aortic unfolding measurement was 103.7±13.9 mm.

**Table 1:** Clinical characteristics of the initial study population.

	Overall (n = 219)	Women (n = 77)	Men (n = 142)	*p*Value
Age (yrs) *	54.2±9.3	56.1±10.2	53.2±8.7	0.0290
BSA (m^2^) *	1.74±0.18	1.57±0.10	1.83±0.11	<0.0001
BMI (kg/m^2^)*	24.2±3.9	23.0±2.7	24.9±4.3	<0.0001
Family history of CHD†	24 (11%)	10 (13%)	14 (9.9%)	0.4792
LVH†	34 (15.5%)	6 (7.8%)	28 (19.7%)	0.0037
Plasma creatinine (mg/dL)*	0.9±0.2	0.7±0.1	1±0.1	<0.0001
Hypertension†	56 (25.6%)	16 (20.8%)	40 (28.2%)	0.2313
Diabetes†	33 (15.0%)	5 (3.5%)	28 (19.7%)	0.0090
Dyslipidemia†	18 (8.2%)	7 (4.9%)	11 (7.7%)	0.7294
Smoking status†	129 (58.9%)	10 (7%)	119 (83.8%)	<0.0001
Aortic unfolding (mm) *	103.7±13.9	98.4±12.9	106.5±13.5	<0.0001

*Data are the mean±standard deviation.

† Data are the number of subjects with %.

*BSA* body surface are, *BMI* body mass index, *CHD* coronary heart disease, *LVH* left ventricular hypertrophy.

There was excellent inter-observer reliability between the two readers (ICC = 0.989; 95% confidence interval, 0.984–0.992; *p*<0.0001] and excellent intra-observer reliability between the first and second evaluations (ICC = 0.991; 95% confidence interval, 0.988–0.993; *p*<0.0001).

In the 2-year follow-up, only 1 male patient who had aortic unfolding of 119 mm underwent revascularization 10 months after CT scan. Instead of major adverse cardiovascular events, we used the CAC score to examine the association between aortic unfolding and atherosclerosis.

### Aortic Unfolding: Correlation with Age

**Figure 2:**
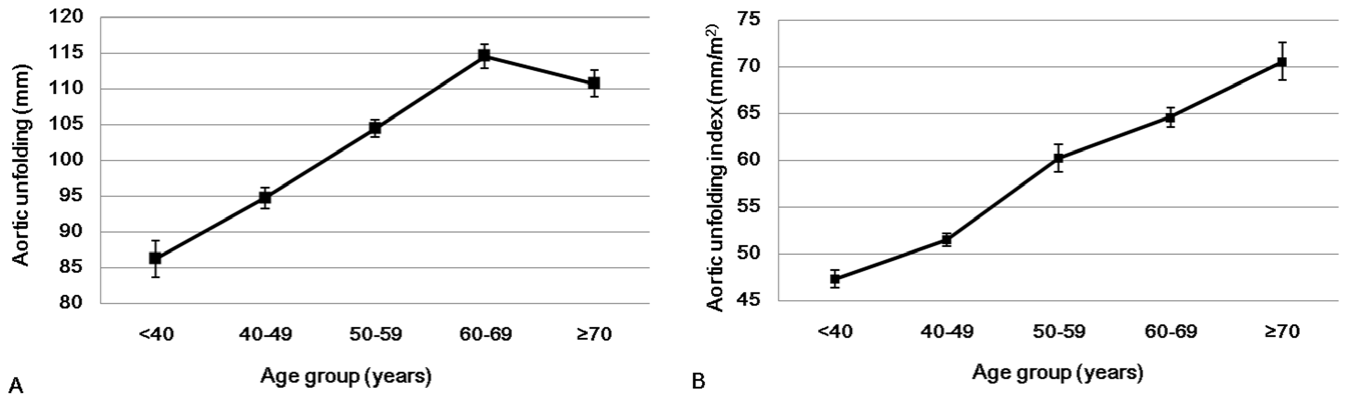
Relationships of aortic unfolding (A) and the aortic unfolding index (B) with age. Although aortic unfolding index increased throughout age decades, aortic unfolding index in men showed plateau at ≥70 years.

Aortic unfolding as measured using non-contrast cardiac CT was correlated with age (β = 0.83, *p*<0.0001). When aortic unfolding was standardized to BSA, the resulting aortic unfolding index (mm/m^2^) was also correlated with age (β = 0.66, *p*<0.0001) (Table 2). Although the aortic unfolding measurement did not increase with age over 60 years old for decrease in BSA decompensated the aortic unfolding, the aortic unfolding index increased throughout all age decades (Fig. 2). A Jonckheere-Terpstra test performed on the trend of gradual increase in AU and AUI with increasing age decades was statistically significant (*p*<0.0001).

**Table 2:** Aortic unfolding and the aortic unfolding index by gender and age.

	Aortic unfolding index (mm/m^2^)*			Aortic unfolding (mm)*		
Age (yrs)	Overall (n = 219)	Women (n = 77)	Men (n = 142)	Overall (n = 219)	Women (n = 77)	Men (n = 142)
<40 (n = 12, m/w = 9/3)	48.0±3.0	50.0±2.3	47.4±3.0	86.2±9.0	75.3±4.9	89.9±6.8
40–49 (n = 51, m/w = 37/14)	53.0±4.7	52.6±5.0	54.0±4.0	94.7±10.5	86.1±5.1	98.0±10.3
50–59 (n = 97, m/w = 62/35)	60.3±6.5	61.8±5.9	59.4±6.7	104.4±12.5	96.8±9.4	108.8±12.0
60–69 (n = 47, m/w = 31/16)	66.2±7.3	68.1±6.0	65.2±7.8	114.5±11.5	110.0±10.4	116.9±11.5
≥70 (n = 12, m/w = 3/9)	72.2±7.2	74.4±7.1	65.7±1.7	110.7±6.5	111.0±6.5	109.9±7.9

*Data are the mean± standard deviation.

### Factors Associated With Aortic Unfolding

Univariate analyses revealed significant associations between aortic unfolding and male gender (β = 7.96, *p*<0.0001), age (β = 0.83, *p*<0.0001), BSA (β = 22.08, *p*<0.0001), BMI (β = 1.08, *p*<0.0001), LVH (β = 6.12, *p*<0.0001), plasma creatinine (β = 18.59, *p* = 0.0007), hypertension (β = 12.97, *p*<0.0001), smoking status (β = 5.05, *p* = 0.0069), and calcium score (β = 0.03, *p*<0.0001). Diabetes, dyslipidemia, and family history of CHD were not significantly associated with aortic unfolding and were removed from the multivariate regression analysis. Age, BSA, and hypertension were independent factors associated with aortic unfolding (β = 0.85, 26.70, and 6.35, respectively; all *p*<0.05). Table 3 presents the result of the multivariate regression analysis.

**Table 3:** Multiple regression analysis of association of clinical factors with aortic unfolding.

Predictors	Parameter estimates	Standard error	*p*Value
Male gender	1.67	2.69	0.54
Age (yrs)	0.85	0.08	<.0001
BSA (m^2^)	26.70	6.63	<.0001
BMI (kg/m^2^)	0.22	0.21	0.29
LVH	0.48	2.05	0.82
Plasma creatinine (mg/dL)	−1.97	5.24	0.71
Hypertension	6.35	1.61	0.0001
Smoking status	0.40	1.88	0.83
Calcium score	0.006	0.005	0.19

*BSA* body surface are, *BMI* body mass index, *LVH* left ventricular hypertrophy.

The 10-year CVD risk was significantly correlated with aortic unfolding (correlation coefficient = 0.62; *p*<0.0001). After adjusting for age and sex, 10-year CVD risk was correlated with aortic unfolding (β = 0.159, *p*<0.0001).

### Relationship between Aortic Unfolding and Calcium score

Stepwise regression analysis was used to control for confounding factors and included aortic unfolding, age, gender or aortic unfolding, 10-year CVD risk as independent variables. The association between aortic unfolding and CAC score was significant after adjusting for age and gender (β = 1.89, *p* = 0.017) or for Framingham risk score (β = 2.83, *p*<0.001).

## Discussion

Aortic unfolding measured on non-enhanced cardiac CT showed excellent inter- and intra-observer agreement and was correlated with age. Age, BSA, and hypertension were independent factors associated with aortic unfolding. Aortic unfolding was positively associated with coronary atherosclerosis burden and that has not been previously described.

Aging is an important determinant of cardiovascular risk and is associated with a number of changes in the structure and function of the cardiovascular system, including the large arteries [23], [24]. Age-associated vascular structural remodeling includes an increase in vascular intimal thickness along with lumen dilatation and vessel stiffening [6]. Dysregulation of the balance between collagen and elastin, as a result of stimulation by an inflammatory milieu or hypertension, leads to the overproduction of abnormal collagen and reduced quantities of normal elastin, thus contributing to vascular stiffness [25], [26]. Aortic stiffening with age leads to an increase in pulse pressure and isolated systolic hypertension [14]. Elevated systolic BP promotes LVH and ventricular stiffening, leading to increased LV oxygen requirements, diastolic dysfunction, and heart failure [24]. In addition, low diastolic pressure reduces coronary blood flow, aggravating the situation and predisposing to ischemia [8], [24].

Human cross-sectional studies have found that wall thickening and dilatation are prominent structural changes of the large elastic arteries during aging [27]. The aortic dimensions can be measured using ultrasound, invasive angiography, CT, or cardiac MRI [2]. All of these modalities have diagnostic value, but CT is the mainstay of the evaluation because of its accuracy, reproducibility, speed, simplicity, and true three-dimensional capability [28]. Recent studies utilized CT or MRI to measure aortic unfolding and revealed the relationship between aortic unfolding and aging, body weight and blood pressure. However, correlation between aortic unfolding with other cardiovascular risk factors and coronary atherosclerosis burden has not yet been fully determined. [7], [29], [30]. The strength of our study is that the measurement of aortic unfolding is simple and it does not need contrast material. Furthermore, aortic unfolding is easy to measure and showed high reproducibility.

Vascular stiffness can be measured using several non-invasive methods, including PWV and AI [4], although these two are influenced by BP and heart rate [3], [9], [10]. Redheuil *et al*. [15] reported that age-related alterations in aortic arch geometry measured on MRI, specifically aortic unfolding, are related to functional aortic alterations, such as decreased aortic distensibility, augmented aortic arch PWV, and increased LV mass, in individuals without overt cardiovascular disease. Therefore, aortic unfolding measured on CT may provide an indication of chronic changes in aortic stiffness that is free from the effects of temporary changes in heart rate and BP during the examination. This advantage makes aortic unfolding measurements worthy of further investigation. While aortic stiffness can be calculated in terms of distensibility at a regional level using coronary angiography, echocardiography, or MRI [31], these techniques have the limitations of requiring more resources, including both end-systolic and end-diastolic images, to calculate distensibility and providing a relatively smaller scale of measurement, making measurement error a relatively serious issue. Aortic unfolding offers a greater measurement scale, giving it an advantage over aortic distensibility. Aortic unfolding measurements can be made in addition to CAC scoring on non-contrast cardiac CT and could enable further stratification of future cardiovascular risks without additional cost, as the CAC score is a strong independent predictor of coronary events [32].

A salient finding of this study is that aortic unfolding measurements from the same non-contrast cardiac CT as used for determining CAC scores were strongly related to age, BSA, and hypertension, even after adjusting for confounding factors. According to the current studies with CAC scan[12] and MRI [15], aortic dilation and the change in geometry contribute differentially to the age, BSA and hypertension. Aortic unfolding increased with age, plateauing at 60 years of age. The increasing tendency of aortic unfolding according to aging were more prominent in younger individuals (<60 years), similar to reported tendency of AI, whereas that of aortic PWV is more marked in individuals older than 50 years [17] Therefore, aortic unfolding and the central AI may be more sensitive markers of arterial aging in younger individuals. Aortic unfolding measurements were greater in men than in women, but the aortic unfolding index was similar between men and women because it takes into account the difference in BSA between men and women. Although aortic unfolding index increased throughout age decades, aortic unfolding index in men showed plateau at ≥70 years. Increasing tendency of aortic unfolding index at ≥70 years reflects the female pattern, and it may be due to dissimilar gender composition (0.3 male per female). We found that 10-year CVD risk is positively associated with aortic unfolding. That probably reflected the cumulative influence of age, gender and hypertension.

Another important finding is significant association between aortic unfolding and subclinical atherosclerosis burden reflected by CAC score, a valuable surrogate marker of coronary atherosclerosis and cardiovascular risk. It is noteworthy that aortic unfolding was related with CAC score independently of conventional cardiovascular risk factors, as reflected by age, gender and Framingham risk score.

In this study, relatively high percentage of LVH (19.7%) was shown in men. LVH is associated with age, BP, obesity, valve disease and MI [33]. Male subjects had relatively high percentage of hypertension (28.2%) and diabetes (19.7%) although no one had concurrent valve disease. That can influence the prevalence of LVH.

Some limitations of this study should be noted. First, this was a single-center, retrospective, cross-sectional study. Second, our study was limited to asymptomatic subjects from a health-screening program and there was short term follow-up, making it difficult to form conclusions about cardiac events. Therefore, we recommend that our findings be validated in a larger cohort with longer duration. In addition, geographic and racial differences in calculating cardiovascular risks were not considered in this study. More than 99% of Framingham participants are of European descents and original Framingham equations overestimate the risk in some population such as Chinese and Danish [34], [35]. However, there was no reports available regarding recalibration of the Framingham risk score to correct the overestimation in Korean cohort. Finally, we assessed aortic unfolding using by 2D measurements. Recent studies evaluated aortic geometry using aortic segmentation methods with non-contrast cardiac CT, but such measurement requires longer scan range (from the top of the aortic arch to the level of the diaphragm) and additional 3D reconstruction [29], [30], [36]. In this study, whole aortic arch was not included in the coronary calcium scan. Also, making the centerline of aorta through 3D reconstruction to measure the length, width and curvature of the aortic arch has practical constraints. Thus, we think our measurement may be a simple way to measure aortic unfolding reliably on CAC scan without further radiation exposure. However, further studies regarding the relationship between our methods of measurements and those using 3D segmentation algorithms are needed.

## Conclusion

In conclusion, aortic unfolding defined by measuring aortic width was a reproducible and practical method with non-contrast cardiac CT and associated with age, BSA, and hypertension. CAC score, a well-established surrogate marker of cardiovascular disease is positively associated with aortic unfolding. Further study to evaluate aortic unfolding as a potential predictor of cardiovascular risk is warranted.
